# Modern yeast development: finding the balance between tradition and innovation in contemporary winemaking

**DOI:** 10.1093/femsyr/foac049

**Published:** 2022-10-18

**Authors:** Jennifer M Gardner, Lucien Alperstein, Michelle E Walker, Jin Zhang, Vladimir Jiranek

**Affiliations:** Department of Wine Science, School of Agriculture, Food and Wine, The University of Adelaide, PMB1, Glen Osmond 5064, South Australia, Australia; Department of Wine Science, School of Agriculture, Food and Wine, The University of Adelaide, PMB1, Glen Osmond 5064, South Australia, Australia; Department of Wine Science, School of Agriculture, Food and Wine, The University of Adelaide, PMB1, Glen Osmond 5064, South Australia, Australia; Department of Wine Science, School of Agriculture, Food and Wine, The University of Adelaide, PMB1, Glen Osmond 5064, South Australia, Australia; Department of Wine Science, School of Agriculture, Food and Wine, The University of Adelaide, PMB1, Glen Osmond 5064, South Australia, Australia; Australian Research Council Training Centre for Innovative Wine Production, Urrbrae 5064, South Australia, Australia

**Keywords:** yeast, wine, oenological fermentation, industry survey

## Abstract

A key driver of quality in wines is the microbial population that undertakes fermentation of grape must. Winemakers can utilise both indigenous and purposefully inoculated yeasts to undertake alcoholic fermentation, imparting wines with aromas, flavours and palate structure and in many cases contributing to complexity and uniqueness. Importantly, having a toolbox of microbes helps winemakers make best use of the grapes they are presented with, and tackle fermentation difficulties with flexibility and efficiency. Each year the number of strains available commercially expands and more recently, includes strains of non-*Saccharomyces*, strains that have been improved using both classical and modern yeast technology and mixed cultures. Here we review what is available commercially, and what may be in the future, by exploring recent advances in fermentation relevant strain improvement technologies. We also report on the current use of microbes in the Australian wine industry, as reported by winemakers, as well as regulations around, and sentiment about the potential use of genetically modified organisms in the future.

## Teaser

The number of commercial yeasts available and used by winemakers is ever expanding as are techniques to develop them; the authors review these and survey Australian winemakers for opinion and use.

## Abbreviations

GM: Genetic Modification
GMO: Genetically Modified Organism
GRAS: Generally Regarded as Safe
QTL: Quantitative Trait Loci
MNNG: methylnitronitrosoguanidine
EMS: ethyl methanesulfonate

## Introduction

Yeasts, primarily *Saccharomyces cerevisiae*, are responsible for many of the processes, including alcoholic fermentation, that transform grape must into wine by modification of aroma and flavour through major changes in chemical composition (Swiegers and Pretorius 2005). Although *S. cerevisiae* has long been considered the main workhorse of fermentation, being almost always the dominant species towards the end of fermentation, other species are also present (Fleet 1993, Pretorius 2000, Goddard 2008). A recent flurry of research has also revealed their important impacts on wine flavour and aroma (Anfang et al. 2009, Gobbi et al. 2013, Loira et al. 2015, Varela et al. 2017, Hranilovic et al. 2018, Rollero et al. 2018, Lin et al. 2022). Whilst traditional fermentation has relied on autochthonous yeasts (that is, those occurring naturally in the environment), pure cultures of *S. cerevisiae* have been available to winemakers since the 1960 s (Lodolo et al. 2008, Chambers and Pretorius 2010). However, in recent times an increasing number of non-*Saccharomyces* strains have also become available. Inoculating wine with pure cultures of yeast has become standard industry practice for more reliable, reproducible and timely wine fermentations as well as for specific organoleptic or processing properties (Chambers and Pretorius 2010, Divol and Bauer 2010, Stewart 2016).

Demand for more yeasts with greater robustness or specific impact on wine composition remains strong. As an alternative to traditional environmental isolation approaches, the field of modern yeast improvement is rapidly evolving, with many new techniques being developed, including those involving genetic manipulation. In general, research in this area has avoided techniques considered genetic modification, or else has only used these as a ‘proof-of-concept’. In this way, the array of phenotypes or new capabilities of strains is ever expanding. Embracing these technological developments affords winemakers the opportunity to address new issues such as the viticultural impacts of climate change or changing consumer demands, with speed and flexibility while maintaining or improving wine quality. Excitingly the forward thinking winemaker realises that the use of these new technologies does not preclude the parallel use of traditional methods that work well and maintain a sense of historical relevance. Instead a balance between the old and the new allows winemakers to address modern challenges and produce fantastic and unique wines under their guidance. Perhaps in some cases also contributing to the wine's individual identity.

As mentioned, the number of *S. cerevisiae* and other species of yeast isolated from various environmental niches or bred for specific organoleptic and processing properties continues to grow. Non-exhaustive online searches reveal over 200 different yeasts available to winemakers in Australia alone (Table S1). Almost one fifth of these are derived from improvement technologies including breeding, mutagenesis, hybridisation and directed evolution. Further, several genetically modified cisgenic and transgenic *S. cerevisiae* strains can be used in other jurisdictions but currently are not permitted in Australia under legislation of the Office of the Gene Technology Regulator (OGTR 2001) and Food Standards Australia New Zealand (FSANZ 2019).

## Improving yeast through genetic manipulation

**Figure 1.**
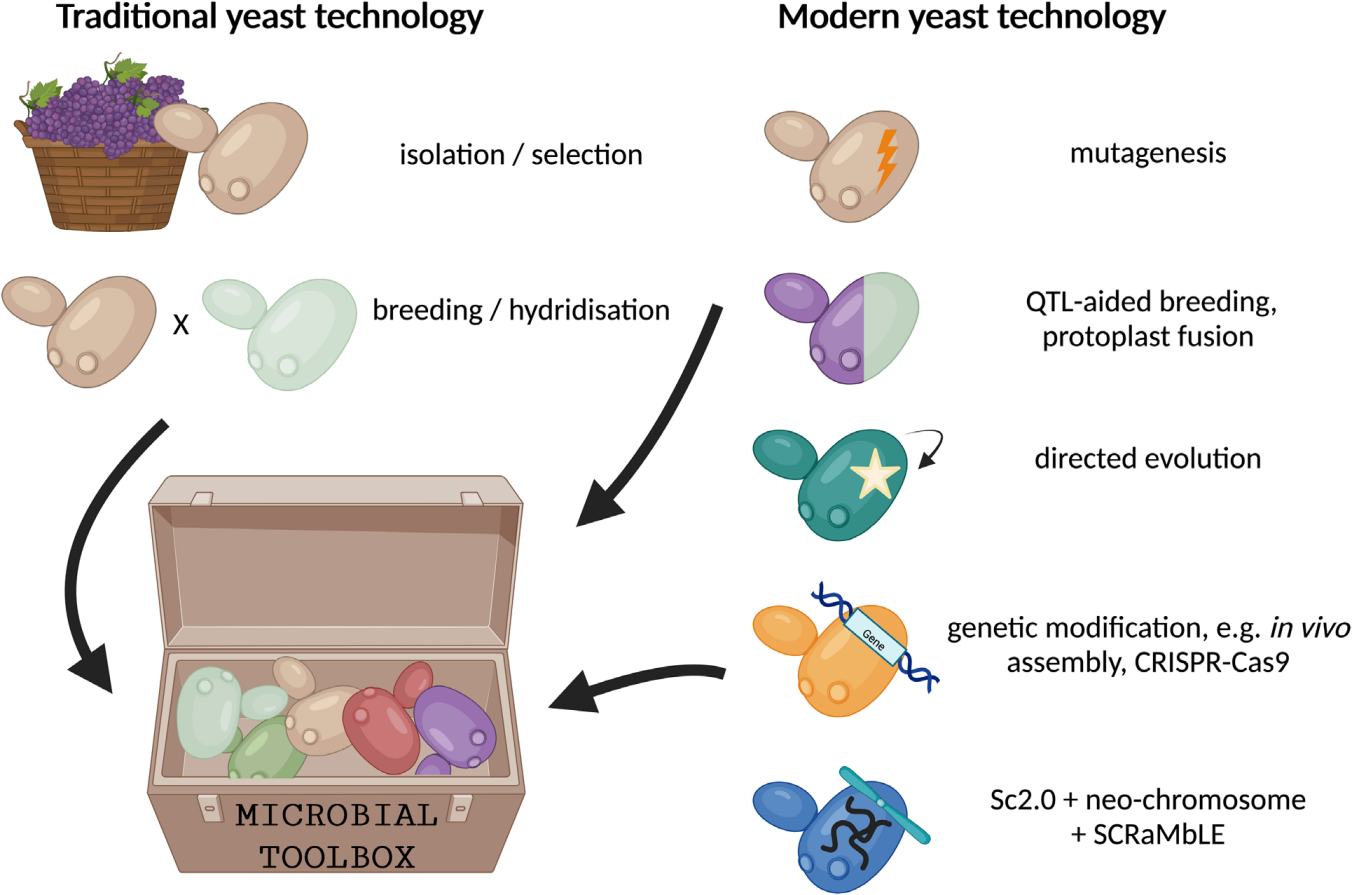
Traditional and modern yeast technologies to produce yeasts for the wine industry. Created with BioRender.com.

Many strategies exist to produce new strains of yeast for the winemaker's toolkit (Fig. 1). These include traditional techniques such as breeding, hybridisation (breeding between different species) and mutagenesis as well as modern techniques that rely on the twenty-first century molecular genetic technique, genetic modification (GM). There are also techniques that might objectively be called GM, particularly by consumers, but depending on legislation of a particular region or country, may in fact not be considered so.

Using these strategies, many advances have been made in the quality and properties of wine yeast available to winemakers. However, winemakers are not always quick to accept these new yeasts—winemaking is steeped in tradition and when marketing wine there is a delicate balance between innovation and novelty versus expectation and tradition (Barber et al. 2020). Even if innovative solutions exist for the winemaker, utilising them may not align with the winery's marketing plan or consumer expectations. Thus, improved wine quality or specific attributes from a particular technology will not necessarily result in greater profit for the winemaker if such changes alienate their customers. This may partly explain why improved yeasts have historically being sought through isolation first, followed by the non-GM genetic techniques of breeding and mutagenesis (Alperstein et al. 2020).

While slow on the uptake compared to the sake (Kitagaki and Kitamoto 2013) and brewing (Gibson et al. 2017, Bonatto 2021) industries, which have adopted purpose-bred yeasts for enhanced aroma/flavour profiles, winemakers are beginning to embrace modern yeast technologies. For example, according to the 2019 Australian Wine Research Institute's survey of winemaking practices, over ten % of yeast used by Australian winemakers in 2019 were derived from modern yeast-breeding techniques (Nordestgaard 2019). Also, our 2021 survey revealed that winemakers are increasingly interested and accepting of yeast made via purposeful genetic modifications, with 53% indicating that genetically modified yeast should be available in Australia (see below). This shift in perception supports the value in future research in this area. However, since only 12% of winemakers thought consumers are ready to accept wines made with GM yeasts, it is clear that hesitancy still exists.

## When genetically modified organisms aren't genetically modified organisms

Different legislation around the world pertaining to the use and consumption of genetically modified organisms (GMOs), and even what constitutes a GMO, has resulted in research and implementation in this area being increasingly divided. For instance, the largest wine-producing regions in the world, Italy, France and Spain, are part of the European Union where there is a significant anti-GMO sentiment and stringent regulation of GMOs in agriculture (Turnbull et al. 2021). In contrast, the market in the United States of America, the world's fourth largest producer of wine, where GM yeasts are legal and available, alcoholic beverages produced with transgenic GM yeast are available to consumers (Denby et al. 2018). For instance, start-up businesses like Berkeley Yeast (Oakland, CA, USA) are actively developing GM yeasts for use in the wine and beer industries with a focus on improving organoleptic properties of fermented beverages.

In Australia, the fifth-largest wine producer in the world, GM regulation falls somewhere between the relaxed US example and the more stringent European model. Some GM crops are grown and in some cases growers must be licenced and follow strict regulations (OGTR 2001). Under the 1999 Food Standards Australia New Zealand Standard 1.5.2 (FSANZ 2019), any GM food must undergo a pre-market safety assessment before approval. GM microorganisms, including yeast, are not approved for human consumption and are therefore not allowed for use in winemaking (FSANZ 2019). However, recent definitional changes to the Australian Gene Technology Regulations 2001 (OGTR 2001); https://www.legislation.gov.au/Details/F2020C00957) further clarify what is legally considered GM. Schedule 1, Regulation 5 of the GTR lists a number of instances that could be applicable to yeasts, where certain genetic modifications would not legally be considered GM and therefore may be allowed for use in wine production. Specifically, Schedule 1, Item 4 allows the use of CRISPR-Cas9 technology (described below) provided ‘a nucleic acid template was not added to guide homology-directed repair’. Thereby non-homologous end joining, where the host organisms DNA repair mechanism is used may qualify (DiCarlo et al. 2013). Further, Schedule 1, Item 9 may allow for breeding GM yeasts where back-crossing (outbreeding) removes any GM-derived molecular machinery but leaves behind the changes to the nucleic acid. Item 6 allows for transfer of genetic material from within the same species, which may essentially allow genes from one yeast to be transferred to another via genetic technologies directly rather than by traditional breeding. Furthermore, under Schedule 1A ‘Techniques that are not gene technology’ (Regulation 4) Item 10 includes the description; ‘A natural process, if the process does not involve genetically modified material’, which could be interpreted as permitting technologies allowing for self-cloning yeast including CRISPR-Cas9. Protoplast fusion is also listed as a technique not considered gene technology in this same Schedule in Australia, however, it is in Europe. The Direction 2001/18/EC of the European Parliament and Council 2001 classifies a GM organism as ‘an organism, with the exception of human beings, in which the genetic material has been altered in a way that does not occur naturally by mating and/or natural recombination’ (of which protoplast has not been shown to occur naturally) and furthermore specifies the inclusion of ‘cell fusion (including protoplast fusion)’ as a technique considered GM. Certainly the regulation of foods produced using GM organisms is very important, and regulatory agencies around the world differ as to how the use of these organisms are assessed, including their classification and safety (Hanlon and Sewalt 2021). When regarded as a processing aid and where no trace of the organism is left in the final product, some countries do not require these products to be labelled as GM (Hanlon and Sewalt 2021). This may certainly apply to GM organisms used in wine, but would seemingly require testing of products to prove no live organisms (or perhaps also DNA) remain.

## Traditional yeast strain improvement techniques

### Isolation/selection

The primary avenue for provision of novel yeast strains for winemakers, whether *S. cerevisiae* or non-*Saccharomyces*, has long been isolation from the environment or directly from active fermentations. Most wine yeast strains have been isolated from uninoculated fermentations, where yeasts from the vineyard or winery equipment have produced a desirable outcome through fermentation. Yeast have also been isolated from other environmental niches including bark, flowers, fruit and insects, some of which may be important reservoirs for overwintering yeast (Stefanini et al. 2012, Madden et al. 2018, Di Paola et al. 2020). Such isolates are systematically evaluated for their potential as wine fermentation starter cultures. Typically, this involves analysis of the fermentation performance when exposed to the typical stressors of wine fermentation such as high sugar (200–300 g L^−1^), low nutrient availability (assimilable nitrogen, expressed here as free amino nitrogen, often < 200 mg FAN L^−1^), ethanol (10%–18% v/v), anaerobiosis, suboptimal temperatures (≤12°C), low pH (2.8–3.8) and the presence of sulfur dioxide (up to 60 mg L^−1^ free, 300 mg L^−1^ total). In turn, the flavour and aroma profile of the resulting wines is also analysed, with particular attention on the absence of undesirable compounds such as hydrogen sulfide, volatile acidity and phenolic aromas and/or the production of desirable sensory impacts. More recently, compatibility of yeast with lactic acid bacteria, necessary for undertaking malolactic fermentation, is also considered as important (Bartle et al. 2019).

While there are many strains of yeast suitable for a broad range of varieties and styles of wine, most are marketed and used solely for a specific grape variety or style of beverage (Supplementary Table 1). For example, strains that produce higher concentrations of particular aromas are favoured for certain wine styles, whereas ‘non-aromatic’ yeasts, which have less of a flavour impact are used where a winemaker wants to express the primary flavours and aroma characteristics associated with the grapes. As mentioned, non-*Saccharomyces* strains have also become available from commercial suppliers and most are promoted as ways to impart distinct characteristics to wine. For instance, *Lachancea thermotolerans* produces elevated lactic acid, however, this may only suit particular styles of beer (Osburn et al. 2018) or wine (Vaquero et al. 2020, Hranilovic et al. 2021) and may impact the success of malolactic fermentation (Snyder et al. 2021). Similarly, other non-*Saccharomyces* such as *Pichia kluyveri*, capable of increasing aromatic thiol concentrations (Anfang et al. 2009), may only be relevant to particular varieties that have higher concentrations of aromatic thiol precursors, such as such as Sauvignon blanc (Jeffery 2016). Winemaker interest in microbes with a bioprotective capability has also attracted much attention lately, with some products already on the market. For instance, Zymafore^®^ KHIO*^MP^* (Laffort, Bordeaux, France) is a *Metschnikowia pulcherrima* selected for its ability to grow at very low temperatures. Whilst one of its main uses is to protect fruit prior to fermentation, for instance in pre-fermentation cold soak, the yeast may also be useful during transport from the vineyard to the winery. The list of non-*Saccharomyces* species currently of interest continues to increase, and includes *Candida railenensis, Debaryomyces hansenii, D. vanriji, Hanseniaspora uvarum, H. vineae, Kazachstania gamospora, Metschnikowia pulcherrima, Nakazawaea ishiwadae, Pichia fermentans, Rhodotorula mucilaginosa, Schizosaccharomyces pombe, Starmerella bacillaris, Torulaspora delbrueckii, Williopsis saturnus*, and *Zygotorulaspora florentina* (Jolly et al. 2014, Pretorius 2022).

Commercial yeasts, usually sold as active dried cultures, can be used as the sole inoculum, historically the most common method. However, recently more winemakers are using two or more strains as a mixed or co-culture fermentation—certainly an increasing number of commercial products are becoming available specifically for this practice. Inoculation of mixed culture fermentations can be simultaneous or staggered (‘sequential’). Typically, the less alcohol-tolerant or competitive strain, such as most non-*Saccharomyces*, is inoculated first and allowed time to proliferate and impact wine composition, before a more robust *Saccharomyces* strain is introduced some days later to ensure fermentation completion. There is also the option to inoculate cultures at different ratios allowing strains with a higher initial population to be more prevalent at the beginning of fermentation, yet at the same time allowing the whole population access to nutrients that may become depleted in the latter stages of fermentation. Indeed some commercial yeast suppliers provide active dried cultures as mixed species preparations in an already optimized ratio, e.g. Viniflora Melody (Chr. Hansen, Denmark) or Zymaflore® Égide (Laffort, Bordeaux) (Supplementary Table 1).

## Modern yeast strain improvement techniques

### Yeast hybrids

Yeast can exist with a single or multiple copy of chromosomes (e.g. haploid, diploid, tetraploid, aneuploid), referred to as ploidy. Haploid yeasts are able to mate with relative high frequency and produce offspring with novel (mixed) genomes and, therefore, fermentation characteristics (Fukuda 2020, Tuite 1991). As well as *S. cerevisiae*, closely related species of *Saccharomyces* yeast can breed albeit rarely, whether spontaneously or in a controlled manner in the laboratory, to form interspecific hybrids with novel fermentation characteristics (Belloch et al. 2009, Morales and Dujon 2012, Bellon et al. 2013, Steensels et al. 2014, Bellon et al. 2015, Krogerus et al. 2018, Sampaio 2018, Sipiczki 2018, García-Ríos et al. 2019). Whilst the similarity of *S. cerevisiae* to the other yeast within the *Saccharomyces* clade allows for this interspecific hybridisation, the hybrid genome is often unstable. Polyploid ‘mismatching’ of chromosomes leads to variable loss of the parent genomes in the newly constructed hybrid, thereby creating uncertainty in the outcome and requiring extensive evaluation of hybrids (Belloch et al. 2009, Bellon et al. 2011, Dujon and Louis 2017). Commercial examples exist of yeast strains that are naturally occurring interspecific hybrids and/or deliberately bred strains. *S. pastorianus*, a *Saccharomyces eubayanus* x *S. cerevisiae* hybrid is typically used in lager-style beer production, and can be found in nature (Sampaio 2018), or as the product of deliberately crossing the parental strains (Krogerus et al. 2015). *S. bayanus*, a hybrid of *S. cerevisiae, S. uvarum* and *S. eubayanus* (Libkind et al. 2011, Nguyen et al. 2011, Pérez-Través et al. 2014), having the ability to tolerate high alcohol and sugar contents, low pH and temperatures, makes it favoured for cold temperature fermentation of white wine (Eglinton et al. 2000), ice wine (Kelly et al. 2018) and ice cider (Bedriñana et al. 2017), with the resultant aroma profile being different to that of *S. cerevisiae*.

Other hybrid strains have been created in laboratories for winemaking (Bellon et al. 2011), such as AWRI 1501, an *S. cerevisiae* x *S. paradoxus* strain capable of producing distinctive wine with high concentrations of aromatic esters, including 2-methylbutyl acetate (Blazquez Rojas *et al*. ) and AWRI 1572, a *S. cerevisiae* x *S. uvarum* hybrid, which has a low acetic acid profile in high sugar (ice wine) fermentations (Bellon et al. 2015). This strain was further improved using evolution during batch fermentations coupled with selection of isolates with chromosomal rearrangements that may increase fitness (Bellon et al. 2018). Some hybrid strains are available commercially, for instance AWRI 1501 is marketed as AWRI Paragon (AB Biotek). As mentioned, industrial strains are generally difficult to breed using traditional mating techniques as they may be sterile due to a low frequency of mating type switching, if they are capable at all, as well as other genomic complexities. A clever technique of increasing the chance of mating in brewing strains has been to induce the mating type switch using CRISPR-Cas9 (described later), and so successfully generate stable hybrid strains (Krogerus et al. 2021); this technique may be useful when applied to wine strains. Another technique used in this area is genome shuffling via whole genome transfer. This is where genomic DNA from one strain is purified and transformed into a recipient strain where the genomes can recombine. An example of this strategy is the improvement of xylose utilisation and ethanol tolerance in *S. cerevisiae* using genomic DNA from *Pichia stipitis* (Zhang and Geng 2012). In the past this technique had not been routinely explored as the potential for genomic rearrangement is large and extensive genomic sequencing may be required to track changes. However, groups have shown that whole genome transfer results in surprisingly few genomic changes (Stojiljković et al. 2022).

### Directed laboratory evolution

Where yeast strains exhibit some but not all characteristics that make them ideal for alcoholic fermentation, directed evolution, has been demonstrated over almost two decades as a useful tool to enhance their properties (McBryde et al. 2006). A typical experimental setup involves conducting continuous fermentation or multiple rounds of batch fermentation in a stressful environment where variants with beneficial mutations are likely to exhibit a growth advantage, thereby ensuring the offspring of these variants progressively become more prevalent in subsequent generations. For example, yeast may be grown in continuous culture containing ethanol in order to direct evolution towards a more ethanol-tolerant phenotype (Novo et al. 2014). Other targets include optimised fermentation performance, in particular fructose utilization (Walker et al. 2022), increased flux through the pentose phosphate pathway (Cadière et al. 2011), higher alcohol or osmotic stress tolerance (Betlej et al. 2020), reduction of alcohol yield (Tilloy et al. 2014) or increased glycerol (Kutyna et al. 2012) or other secondary metabolite production. To increase the likelihood of obtaining improved strains via directed evolution, some have sought to increase the diversity of the starting/evolving population by performing genome shuffling via spore conjugation or by mutagenesis. The former involves sporulation of the parent strain and then allowing conjugation of gametes. This technique has been successfully used in conjunction with directed evolution to obtain strains with altered sulfate assimilation (De Vero et al. 2011) and increased glutathione production (Mezzetti et al. 2014), with the latter now available commercially (GLUTAFERM One, AEB). Alternatively, a population may be ‘pre-mutagenised’ or mutagenised periodically in an effort to enable or accelerate the appearance of desirable variants.

Directed evolution can be time-consuming, often requiring many hundreds of generations before beneficial phenotypes are fixed in the population. Experimental design is vital to reduce genetic drift as the whole genome is a potential target for mutation. However, it does offer the advantages of maintaining the original background and characteristics of the parent strain, no requirement for prior knowledge about the genetics/biochemical pathway involved, no need for introduction of foreign DNA and not being regarded as GM and thus immediately applicable to industry use.

### Mutagenesis

Chemical and physical mutagenesis strategies have been used to mutate yeast for brewing and winemaking purposes since at least the 1970s (Alikhanyan and Nalbandyan 1971, Molzahn 1977), where ultraviolet light or chemicals including methylnitronitrosoguanidine (MNNG) or ethyl methanesulfonate (EMS) are used to mutate yeast DNA in order to induce a change in genotype and therefore potentially influence phenotype (Steensels et al. 2014). Mutations will occur naturally in any organism, but mutagenizing agents greatly increase the rate of occurrence. In all instances, mutations are almost completely random, thus a suitable method for screening newly created mutants is crucial. Cordente et al. (2009) successfully mutagenized commercial *S. cerevisiae* strain Maurivin PDM using EMS to produce commercial strain Maurivin Platinum. The mutant strain has very similar organoleptic and processing properties to the parent except for greatly reduced hydrogen sulfide production and higher total sulfite production. More recent work in this area using similar methods resulted in the isolation of strains with both reduced hydrogen sulfite and sulfur dioxide (Walker et al. 2021). Further examples of mutagenesis-derived yeast include AWRI/Maurivin's Rosa and Rosa Intense yeast strains, where a strategy to screen for novel mutants that overproduce ‘floral’ aroma compounds 2-phenylethanol and 2-phenylethyl acetate was used (Cordente et al. 2018). Here researchers screened for phenylalanine biosynthetic pathway genes that led to the overproduction of these aroma molecules.

### QTL marker breeding

DNA marker–assisted breeding, also known as Quantitative Trait Loci (QTL) breeding, involves traditional breeding by one of the above-mentioned strategies of two yeasts with opposing phenotypes or traits of interest (e.g. a high and low producer), combined with molecular DNA sequencing to identify key genes and mutations, often referred to as markers, associated with the desirable trait (Liti and Louis 2012, Swinnen et al. 2012). This then allows researchers to screen progeny or indeed other strains for the desirable phenotype by following the linked markers. A QTL change known to provide a yeast species with a particular trait is selected for in the breeding process, with selection confirmed largely by genome sequencing as well as phenotypic analysis. In this manner targeted changes to DNA can be selected, allowing for precise selection of fermentation characteristics such as malic acid degradation. Combined with backcrossing, QTL marker breeding can allow for single, precise changes to be made to a strain with known characteristics. This is potentially advantageous when compared to mutagenesis or traditional yeast hybridisation, where although beneficial mutations are introduced to a yeast strain by these techniques, there may also be unintended changes to the genotype, and therefore phenotype of the yeast. Commercial wine yeast supplier Laffort (Bordeaux, France) has used QTL breeding in the development of six of the 46 strains available in the Australian market (Table S1).

### GM techniques

Publishing of the complete genome sequence of *S. cerevisiae* in 1996 (Goffeau et al. 1996) opened up the possibilities for genetic engineering of this species for both research and industry, including winemaking.

Many researchers have exploited the inherent, rare and very useful trait of homologous recombination in *S. cerevisiae* that repairs double stranded breaks (Kunes et al. 1985). This capability of *in vivo* assembly, also known as homologous integration, allows genomic incorporation of gene cassettes made using classical PCR and cloning techniques after delivery to the nucleus with transformation (Gietz and Woods 2001, Storici et al. 2001). Gene expression in yeasts has many levels of regulation thus modification or indeed addition of specific genes can result in vastly different impacts depending on the level of gene expression. Certainly within a wine context, when considering effects on aroma of metabolites at higher or different concentrations, fine-tuning of expression can be key to the impact on wine quality. Recently, research groups have improved these technologies, making gene expression easier to control with thoughtfully designed systems, such as carefully selected promoters (Decoene et al. 2019) and terminators (Curran et al. 2013).

There are many examples of yeast modified using this classically targeted technology, mostly within *S. cerevisiae*, but tools for non-*Saccharomyces* also exist or are being developed (Varela et al. 2020, Badura et al. 2021). Many have shown excellent potential to address specific fermentation issues and improve wine quality and aroma. Few, however, have made it to market, with strict regulations in place in many countries. Research using these techniques has instead been intended as a ‘proof-of-concept’. Regulations may well change in the future and some companies have a positive outlook evidenced by their release and even offering of custom strain improvement services (Berkeley Yeasts, CA, USA; Ginko Bioworks, Boston, MA, USA). There are a few exceptions including the GM *Saccharomyces cerevisiae* ML01 (Lessaffre Yeast Corporation, Milwaukee, WI, USA), which enjoys the ‘Generally Regarded as Safe’ (GRAS) status from the US Food and Drug and Administration as of 2003 (GRN No. 120) and ‘no objection to the food use’ by Health Canada. ML01 is able to decarboxylate malic acid, a process usually undertaken by lactic acid bacteria during malolactic fermentation. However here, the yeast is capable of undertaking due to the expression of malate permease, originally from *Schizosaccharomyces pombe*, and mleA from *Oenococcus oeni* (Husnik et al. 2006). Likewise, ECMo01 (First Venture Technologies Corp, Vancouver, BC, Canada) is also available for use commercially in permitting countries. It has high urea degradation, and thus reduced potential and risk ethyl carbamate formation, and also received GRAS status (GRN No. 175) in 2006 and ‘no objection’ from Health Canada. It was constructed by over-expression of the *DUR1, 2* genes, which encode urea amidolyase (Coulon et al. 2006).

Yeasts have also been engineered for improved robustness and fermentation efficiency in the wine environment, for instance, by manipulation of genes involved in stress response(s) (Jiménez-Martí et al. 2009). Deletion of a handful of genes has also been shown to result in efficient fermentation in other challenging conditions such as under nitrogen-limited conditions—a very common occurrence in Australian juices (Gardner et al. 2005, Peter et al. 2018, Zhang et al. 2018). The focus of improvement of wine aroma has been a popular target also, for example: through the production of the raspberry ketone, 4-[4-hydroxyphenyl]butan-2-one (Lee et al. 2016), an increase in the formation of ‘fruity’ acetate esters by overexpression of alcohol acetyltransferase (*ATF1*) (Lilly et al. 2000), release of volatile thiols to increase the passionfruit aroma by expression of the *Escherichia coli tna*A gene (Swiegers et al. 2007), increase in total monoterpenes via expression of geraniol synthase from sweet basil (Pardo et al. 2015), and increase in the aromatic compound, linalool, by expressing the *Aspergillus aculeatus rhaA* gene (Manzanares et al. 2003).

Many studies have also used classical targeted genetic manipulation in an attempt to address the complex problem of lowering alcohol production by yeasts. So far, however, only small reductions or concomitant undesirable consequences have been realised (Varela et al. 2012). The most successful approach seems to be the overexpression of the glycerol-3-phosphate dehydrogenase isoenzymes encoded by *GPD1* and *GPD2* to redirect carbon towards glycerol instead of ethanol, plus deletion of *ALD6* (acetaldehyde dehydrogenase) to reduce the undesirable accumulation of acetate (Cambon et al. 2006) and further, overexpression of NADH-dependent 2,3-butanediol dehydrogenase (*BDH1*) to reduce acetoin (Ehsani et al. 2009). More recently a multi-omics approach was used to understand how further modifications, such as deletion of pyruvate decarboxylase (*PDC5*), could be used to enhance the wine aroma profile of these low alcohol producing yeasts by reducing off-flavours (Varela et al. 2018). Also a global transcriptional engineering approach has been used to find another potential gene target (*SPT15*), although the mutant reported in the study exhibited slow growth (Du et al. 2020).

Over the years wine yeasts have been engineered to produce nutraceuticals, or what some consider to be health-promoting compounds. In some cases, the impact of these on human health is a matter of contention, but this will not be covered here. That said, the levels of resveratrol, thought to be antioxidative and anti-inflammatory, amongst other claims, was increased with the expression of the 4-coumarate coenzyme A ligase gene (4CL) from *Arabidopsis thaliana* and the resveratrol synthase gene (RS) from *Vitis vinifera* (Sun et al. 2015). Other targets have been proposed, as reviewed in (Vilela 2019), and include melatonin, serotonin, tyrosol, hydroxytyrosol, caffeic acid, tryptophol, glutathione and trehalose.

The use of microbes to protect wine from spoilage organisms, is a relatively new concept and work on this goal has made use of classical genetic manipulation. Branco and colleagues (Branco et al. 2019) found antimicrobial peptides derived from the glycolytic enzyme glyceraldehyde-3-phosphate dehydrogenase in *S. cerevisiae* are inhibitory to the spoilage yeast *Brettanomyces*/*Dekkera bruxellensis*. Native production of these was, however, too low to be fully inhibitory, so the group increased peptide (saccharomycin) production by overexpression of the *TDH1* and *TDH2/3* genes.

A number of researchers have modernised approaches to genetic manipulation of yeast, moving away from classical targeted genetic manipulation and instead taking advantage of the CRISPR-Cas9 system. Developed by the research teams of Emmanuelle Charpentier and Jennifer Doudna (Doudna and Charpentier 2014) who were awarded the 2020 Nobel prize for Chemistry, the system is particularly useful for altering industrial yeasts as it enables genome editing that is precise and marker free, avoiding the need for antibiotic resistance or auxotrophy rescue markers to select for transformants. It also allows seamless editing of the genome, avoiding the downstream complications of extra and unwanted sequences being left in the genome. As it was discovered in nature with bacteria using the system for millions of years, it is also considered less ‘man-made’. A plethora of improvements to this technique are being reported, including simultaneous multiple edits using gRNA-tRNA array for CRISPR-Cas9 (GTR-CRISPR) (Zhang et al. 2019), where up to eight edits are possible with 87% efficiency.

A number of groups are already using CRISPR-Cas9 technology with wine yeasts. Some of the first sought to decrease urea production and thus reduce the risk of ethyl carbamate accumulation by modification of the arginine permease gene (*CAN1*) (DiCarlo et al. 2013, Vigentini et al. 2017), or to increase rose/honey like aromas by increasing production of phenylethyl acetate (Trindade de Carvalho et al. 2017). Subsequently, others have increased fermentation speed by deletion of the high affinity phosphodiesterase (*PDE2*) (Vallejo et al. 2020), decreased accumulation of unpleasant hydrogen sulfide (Walker et al. 2021), improved fermentation efficiency in low nitrogen fermentations (Lang et al. 2021), improved utilisation of proline, as an alternate nitrogen source not normally metabolised under wine conditions (Luo et al. 2018), and reduced growth rate to encourage growth of competing (but desirable) yeasts through modification of S*ER1* (Lang et al. 2022). This technology is also being used to better understand spoilage organisms and the role of sulfite tolerance in *Brettanomyces* with deletion of the sulfite permease (*SSU1*) (Varela et al. 2020). Furthermore, some groups are beginning to combine traits in a single strain, for example overexpression of glycerol-3-phosphate dehydrogenase and alcohol acetyl transferase 1 resulting in yeasts with increased glycerol and acetate ester production (van Wyk et al. 2020).

Recently, the research community has come together to design and synthesise a completely synthetic version of *S. cerevisiae* (Sc2.0), of which a draft sequence has been reported (Richardson et al. 2017). If successfully completed, this will be the first man-made genome, representing a huge leap in synthetic biology, opening up many possibilities for industrial yeast design. Even though the first sequenced strain of yeast, S288c (Goffeau et al. 1996), is a long domesticated strain and bears many differences to industrial wine yeasts, its sequence has faithfully served as a template for whole genome sequencing of many related yeasts (Borneman et al. 2011, Borneman et al. 2016).

Building upon this, researchers from the Australian Wine Research Institute and Macquarie University have constructed a neo-chromosome of pan-genomic elements unique to 200 industrial or environmental *S. cerevisiae* isolates (Kutyna et al. 2022). Sequences were concatenated to form a 17th chromosome compatible with Sc2.0. A key feature of the design of Sc2.0, and also included in this neo-chromosome, is the capability to introduce genetic diversity with the presence of multiple loxPsym sites, allowing rearrangement of the genome with a technique known as SCRaMbLE (Dymond et al. 2011). This enables multiple inversions and deletions and thus mixing, reordering and deleting parts of the genome. Generation of a pool of genetically diverse isolates in this way followed by selection and screening is akin to an accelerated evolution. Indeed, this has also been used in *Saccharomyces* before, in multiple rounds, known as Multiplex SCRaMbLE Iterative Cycling (MuSIC) (Jia et al. 2018). Other techniques are being developed to further improve SCRaMBLE, such as ReSCuES (Luo et al. 2018), where auxotrophic reporters are used to identify successfully SCRaMbLEd isolates. Use of these techniques in the future could prove extremely fruitful in isolating strains with novel and useful characteristics for winemaking, potentially not found or existing in nature. Excitingly, addition of this extra 17^th^ chromosome to the laboratory yeast strain BY4742 enabled the utilisation of a wider range of carbon sources, similar to industrial yeast (Kutyna et al. 2022). Together with an in-depth knowledge of wine yeast genomes, genetic engineering has become more straight-forward. Not only are we better able to understand the basic biological framework, but also to easily find and target genes and pathways of interest.

Of course, each new improved strain needs not be used in isolation, but possibly with several such organisms in concert. Indeed, this is similar to what happens in reality (grape juice is rarely sterilised), which is why wine fermentations are regarded as a mixed culture. A high level vision of synthetic microbial communities was proposed by Walker and Pretorius (Walker and Pretorius 2022), who describe the advantages and challenges of such multi-strain interactions and how the winemaker might manage or indeed embrace unpredictability in complex systems.

## Use of wine microbes in the Australian Wine Industry: An industry survey

**Figure 2.**
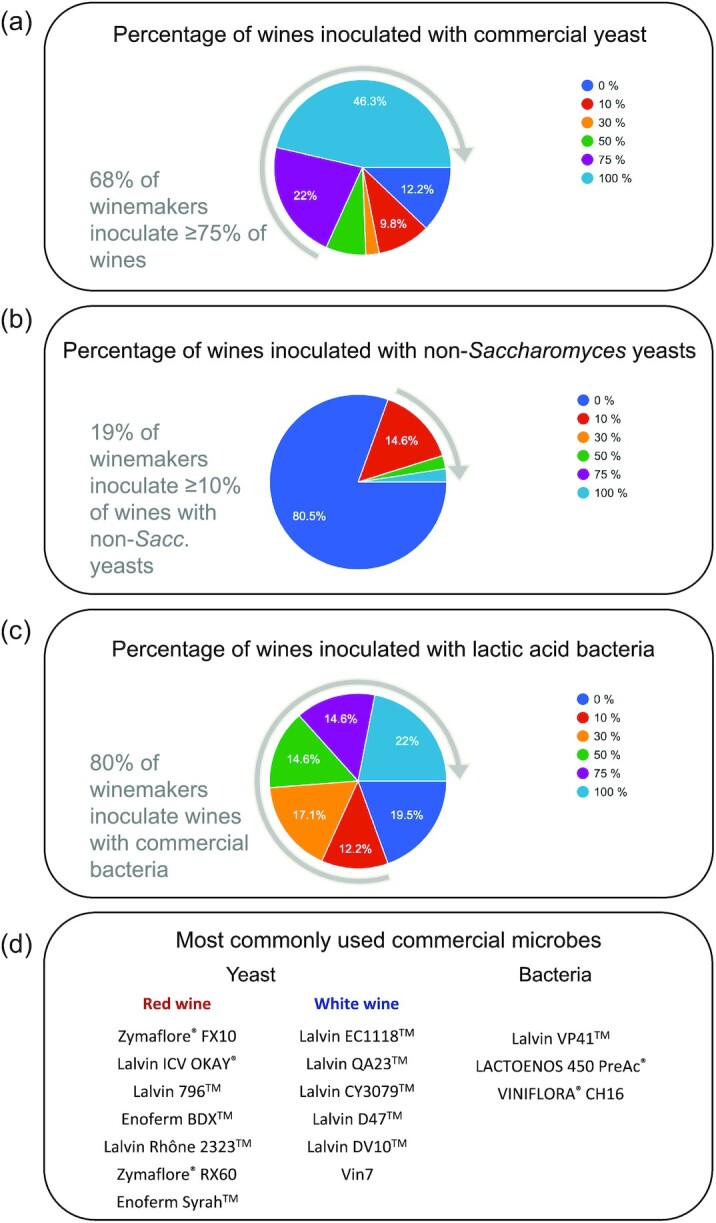
Key results from a Wine Industry Survey conducted in 2021 (full results (Alperstein et al. 2022)). Forty-one winemakers responded and were from an equal distribution of winery sizes and microbial expenditure budgets. The percentage of winemakers that reported inoculation with commercial yeast (**a**), non-*Saccharomyces* yeast (**b**) and lactic acid bacteria (**c**). The most commonly used commercially available yeast and lactic acid bacteria (**d**).

Ultimately, researchers and funding bodies aim to provide useful tools to industry, thus assessment of industry understanding and uptake of such tools is important to guide future research. To this end, we undertook a survey of Australian winemakers and Industry suppliers in 2021 to get an in-depth understanding of the uptake and uses of wine yeasts (Alperstein et al. 2022). Questions were also included on the use of lactic acid bacteria in winemaking, and whilst not reviewed here, this is certainly an area of rapid development. Forty-one Australian winemakers responded to the survey and were from an equal distribution of winery production scales (i.e. <50, 50–1000, 1000–10 000, or > 10 000 tonnes). The survey revealed that all believed wine microbiology affected final wine quality and 90% of respondents also consider research in this field to be highly to very highly important. Use of commercial yeast cultures was very common, with 68% choosing to inoculate 75% or more of their fermentations (Fig. 2a). Overall, winemakers chose not to inoculate 12% of fermentations with commercial yeast, and in almost all cases, larger wineries (>10 000t) chose to inoculate 100% of fermentations. In comparison, a Practices Survey conducted by the Australian Wine Research Institute in 2016 found that on average 3% of red and 6% of white fermentations were not inoculated but relied on the indigenous yeast population, often referred to as ‘wild’ fermentations (Nordestgaard 2019). However, when considering winery size, this increased to 35% in very small wineries (<50t).

In our survey, the main reasons for purposeful inoculation were to ensure process efficiency (including fermentation reliability), suppression of spoilage organisms, improved wine quality (including flavour and aroma) and to contribute to consistency between vintages. The most commonly used yeast strains included Zymaflore FX10, Lalvin ICV-OKAY, Lalvin 796, Enoferm BDX, Lalvin Rhone 2323, Zymaflore RX60 and Enoferm Syrah for reds and for whites, Lalvin EC1118, Lalvin QA23, Lalvin CY3079, Lalvin D47, Lalvin DV10 and Vin7 (Fig. 2d). In comparison to the AWRI Survey, three of the top six yeasts used to ferment red wine (by number of wineries) were the same, being Zymaflore FX10, Enoferm BDX and Enoferm Syrah with the addition of Lalvin RC212 and Lalvin Clos. For the most commonly used yeast for white wine fermentations, four strains were the same, being Lalvin EC1118, Lalvin QA23, Lalvin CY3079 and Lalvin DV10 with the addition of Zymaflore X5 and Zymaflore VL3 in the AWRI survey. Most interestingly, in our survey a large number of strains (over 50) were reported to be in use, including a number of relatively new strains (Table S1). This is excellent evidence that the Australian wine industry is keen to try new yeast strains. For instance, 41% of winemakers have already used Zymaflore FX10^®^ (Laffort, Bordeaux, France), which was first made commercially available in Australia in 2010.

Inoculation with commercial non-*Saccharomyces* strains was reported by 19% of winemakers, with *Torulaspora delbrueckii* and *Metschnikowia pulcherrima* being the most common species chosen. In most (67%) of these non-*Saccharomyces* fermentations, *Saccharomyces* was added sequentially, presumably to ensure completion of fermentation. The main reasons given for the use of non-*Saccharomyces* were to increase wine complexity and introduce unique wine aromas. When asked about what attributes of commercial yeast strains were most important, a wide range of responses were given. The most common (64%) being ‘tolerant to normal stresses encountered during fermentation’ closely followed by ‘ability to ferment efficiently at low temperatures’ (47%), ‘a desirable effect on wine composition and quality’ (47%), and ‘low acetic acid/volatile acidity production’ (44%). Clearly, value placed on some of the fundamental attributes of commercial strains suggests that winemakers are still encountering technical failures during fermentation. This is also reflected in this survey where 86% and 73% of winemakers reported that 5% or more of fermentations required processing changes due to stuck or sluggish alcoholic or malolactic fermentations, respectively. Likewise in the AWRI survey, 3–4% of alcoholic and malolactic fermentations were reported to be problematic between 2011 and 2016.

Our survey results also reveal that a wide variety of yeast attributes are sought. For instance, 25 of the 26 attributes presented were selected as useful (Alperstein et al. 2022). This demonstrates that winemakers want yeasts to contribute to fermentation in many ways and is reflected in the large array of commercial yeasts in use, of which many possess both the basic attribute of sound fermentation and desirable wine aromas, but also additional fit-for-purpose traits, for example, suitability to a particular variety or wine style. Where winemakers chose not to inoculate fermentation and instead rely on the indigenous microbes present, the reasons for doing so were similar to those reported for commercial preparations of non-*Saccharomyces* strains: these being to increase wine complexity, possibly through introduction of unique wine aromas and also as an expression of terrior. Supplementation of fermentation with yeast nutrients was also common with 90% of winemakers reporting use (76% used diammonium phosphate and 59% products mainly composed of inactivated yeasts).

The application of commercial malolactic acid bacteria was much more variable. Overall inoculation is commonly used since 80% of winemakers reported they inoculated at least 10% of fermentation, yet the number of fermentation chosen to be inoculated varied, for instance 22% of winemakers inoculated all fermentation, 12%–15% each inoculated 75, 50, 30, or 10% of fermentation, and 20% inoculated none (Fig. 2c). The most common reasoning behind the choice to inoculate with commercial preparations of lactic acid bacteria was that ‘inoculation is considered a more efficient way to achieve completion of malolactic fermentation’ and ‘inoculation leads to higher quality wine’ and conversely ‘the indigenous population is sufficient’. Surprisingly cost was not a significant factor influencing this decision. Twenty strains were reported in use with the most common being Lalvin VP41^TM^ (Lallemand, Quebec, Canada), Lactoenos 450 PreAc^®^ and Viniflora^®^ CH16 (CHR Hansen, Hørsholm, Denmark) (Fig. 2d). Similar to the attributes of yeast, many are considered as beneficial by winemakers with all 16 attributes being selected at least once. The most common selected were ‘tolerant to the normal stresses encountered during secondary fermentation’ (71%), ‘rapid fermentation rate’ (51%), ‘ferments efficiently in elevated alcohol (>15%)’ (44%) and ‘low acetic acid production’ (42%).

When considering which microbiological factors pose the highest risk to wine quality during processing, winemakers reported that ‘spoilage by undesirable organisms’ (90%), and ‘control of the dynamics of alcoholic (44%) and malolactic (32%) fermentation’ as key. Fewer issues were reported with haze or filtration difficulties caused by microbes (15% and 22%, respectively). A number of strategies are employed during a stuck alcoholic fermentation, most commonly re-inoculation with commercial yeasts, agitation, change of fermentation temperature and cross-inoculation with an actively fermenting must. Similarly stuck malolactic fermentations were treated by raising the temperature, cross-inoculation, direct inoculation with a commercial culture or simply waiting.

Some 44% of winemakers believed that GM yeast should be made available for use in Australia, but only 12% believed consumers would accept their use. From the winemakers’ perspective they believed this was for a wide range of reasons, including that consumers were ‘afraid’ of all GM organisms, and that they already were requesting less additives (and GMOs would just be another). Conversely, winemakers also believed that consumers may accept the use of GMOs in winemaking if they had a better understanding of their positive attributes and if they could taste (and thus appreciate) the difference they might make. About 8% of respondents also noted that the use of GMOs in wine was less invasive than other food industries since yeast are not present in the final product. If novel microbes constructed with new genetic technologies (e.g. CRISPR), which allow modification without the addition of foreign genes were available and they aligned with the winemaker's style, 56% said they would use them. This certainly supports the notion that ‘vintners will continue to be inspired by a custom to innovate through tradition’ (Pretorius 2022) and further that ‘Today's wine stakeholders must be wise enough to learn from the past, smart enough to utilize the present, and imaginative enough to anticipate the future with realism and optimism’. Finally, winemakers were asked which technological advances would be most beneficial to their business. Answers were wide ranging and included microbes with better reliability and novel capabilities, such as lower alcohol production, as well as simple monitoring of microbial type, health and spoilage risks, including during fermentation, in storage vessels and in vineyards.

When we surveyed the five major suppliers of microbes for winemaking in Australia they reported that on average 70% of customers were interested or enquired about new microbial strains, this often being the first question asked. Suppliers reported that these enquiries often had a good translation to sales (∼50%), and was especially increased when these products were available on trial. Some hesitation, however, still remains with the reasons being varied, but included preferring to stick to current traditions, being risk averse and in some cases, because there was little understanding of the new technology.

Uptake of new technology by winemakers was also reported to dramatically increase when industry support was available, involving both ease of access to research data and application support. The most effective method of translation was reported to be face-to-face meetings, particularly when researchers and suppliers presented together and when this was well supported by journal articles, specifically aimed at industry. There were mixed opinions regarding whether clients were particularly interested in the techniques used to generate new strains, which is most likely tied to the (often limited) understanding of these techniques. Understandably clients were focussed on the outcomes new strains offered rather than their origins.

Some 35% of customers were also interested in non-*Saccharomyces* strains and suppliers reported that this has increased since 2019. Uptake of these products was much more likely when protocols involved no increase in processing or cost. For instance, non-*Saccharomyces* and *Saccharomyces* blends were more popular that separate cultures since only a single inoculation is required, even though flexibility of strain choice was limited.

## Summary

Modification of wine aroma and flavour with the use of the microbiome is an option for winemakers to shape wines to match consumer desires or tackle new processing challenges such as those arising from climate change. Lowering the inherent risks associated with vintage variability by opening up options for processing of fruit from what may have historically been labelled as ‘a bad year’ may change the landscape of how wine is both judged and sold. Consumer preference seems to be ever evolving, especially recently, perhaps as an artefact of being easily influenced by rapid marketing, possibly to the recent boom in social media. Certainly, use of different microorganisms is rapid, cheap and flexible in comparison to the multi-year process of replanting or grafting a vineyard and winemakers (at least in Australia) are embracing these as evidenced by the industry survey reported here. Novel strains, in particular those produced utilising genetic manipulation and/or synthetic biology, offer an excellent resource to tackle those winemaking problems not solvable with traditional strains, and further enable introduction of new characteristics not available previously. There is no doubt that a plethora of other techniques for improvement of microbes will become available in the very near future, for example, the unexplored area of introduction of synthetic organelles to microbes (Pretorius 2019). Industry leaders should watch this space carefully, with so many options to exploit, this rapidly moving research area, accelerating diversity and product development in winemaking is an opportunity not to be missed.

## Supplementary Material

foac049_Supplemental_File
